# EGF hijacks miR-198/FSTL1 wound-healing switch and steers a two-pronged pathway toward metastasis

**DOI:** 10.1084/jem.20170354

**Published:** 2017-10-02

**Authors:** Gopinath M. Sundaram, Hisyam M. Ismail, Mohsin Bashir, Manish Muhuri, Candida Vaz, Srikanth Nama, Ghim Siong Ow, Ivshina Anna Vladimirovna, Rajkumar Ramalingam, Brian Burke, Vivek Tanavde, Vladimir Kuznetsov, E. Birgitte Lane, Prabha Sampath

**Affiliations:** 1 Institute of Medical Biology, Agency for Science, Technology, and Research (A*STAR), Singapore; 2 Bioinformatics Institute, Agency for Science, Technology, and Research (A*STAR), Singapore; 3 Department of Biochemistry, Yong Loo Lin School of Medicine, National University of Singapore, Singapore; 4 Program in Cancer and Stem Cell Biology, Duke–National University of Singapore Medical School, Singapore

## Abstract

Epithelial carcinomas are well known to activate a prolonged wound-healing program that promotes malignant transformation. Wound closure requires the activation of keratinocyte migration via a dual-state molecular switch. This switch involves production of either the anti-migratory microRNA miR-198 or the pro-migratory follistatin-like 1 (FSTL1) protein from a single transcript; miR-198 expression in healthy skin is down-regulated in favor of FSTL1 upon wounding, which enhances keratinocyte migration and promotes re-epithelialization. Here, we reveal a defective molecular switch in head and neck squamous cell carcinoma (HNSCC). This defect shuts off miR-198 expression in favor of sustained FSTL1 translation, driving metastasis through dual parallel pathways involving DIAPH1 and FSTL1. DIAPH1, a miR-198 target, enhances directional migration through sequestration of Arpin, a competitive inhibitor of Arp2/3 complex. FSTL1 blocks Wnt7a-mediated repression of extracellular signal–regulated kinase phosphorylation, enabling production of MMP9, which degrades the extracellular matrix and facilitates metastasis. The prognostic significance of the FSTL1-DIAPH1 gene pair makes it an attractive target for therapeutic intervention.

## Abstract (precis)

Exploring the parallels between wound healing and epithelial cancers, Sundaram et al. elucidate the mechanism by which cancer cells hijack the wound healing switch to enhance invasion and metastasis in head and neck squamous cell carcinoma.

## Introduction

There is a dire need for new clinical strategies to manage head and neck squamous cell carcinoma (HNSCC). HNSCC results from uncontrolled squamous cell proliferation in mucosal linings of the upper aero-digestive tract and is the sixth leading cancer by incidence worldwide. High rates of metastasis and aggressive disease progression result in poor prognosis for HNSCC patients; more than two-thirds present with metastatic disease for which palliative care is often the only treatment option (Bhave et al., 2011). Overall median survival in these individuals is between 5 and 9 mo.

Identifying therapeutics that target metastasis is therefore of clinical interest in the management of HNSCC. This necessitates an understanding of the metastatic process in epithelial carcinomas, which has close parallels in wound healing. In healing wounds, keratinocytes proliferate and migrate in a self-limiting manner to achieve reepithelialization. These behaviors are switched off upon completion of wound closure. Keratinocyte proliferation and migration are also hallmarks of epithelial carcinomas; however, dysregulation of these processes generates uncontrolled growth and metastasis (Schäfer and Werner, 2008).

Keratinocyte migratory behavior is governed by a bistable molecular switch (Sundaram et al., 2013). This switch, originally identified in the context of wound healing, controls the context-specific expression of two alternate gene products with opposing functions from a single transcript. In normal skin, the transcript functions as a primary miRNA, which is processed into mature miR-198, an inhibitor of keratinocyte migration. Upon injury, the same transcript functions as an mRNA and expresses the promigratory FSTL1 protein. This switch from miR-198 expression to FSTL1 expression upon wounding facilitates temporal migration of keratinocytes and wound reepithelialization (Sundaram et al., 2013).

The similarities in keratinocyte behavior between wound healing and epithelial carcinoma led us to hypothesize that the miR-198/FSTL1 switch might be involved in progressive HNSCC. Here we show that in HNSCC, persistent FSTL1 translation occurs with concomitant miR-198 down-regulation, signifying a defect in the switch. This defect promotes metastasis by activating parallel pathways involving DIAPH1, a promigratory target of miR-198, and FSTL1. FSTL1 interacts with Wnt7a and antagonizes its repression of extracellular signal–regulated kinase (ERK) phosphorylation. ERK phosphorylation stimulates MMP9 expression, which promotes extracellular matrix degradation and metastasis. Simultaneously, the lack of miR-198 relieves repression on DIAPH1, an actin nucleator that stimulates lamellipodia formation and drives polarized cell migration. This effect is enhanced through DIAPH1-mediated sequestration of the negative regulator Arpin. This two-pronged pathway is activated by epidermal growth factor (EGF), which hijacks the miR-198/FSTL1 switch in favor of FSTL1 and steers the cells toward metastasis.

## Results and discussion

### EGF-driven microcircuitry hijacks the wound-healing switch

**Figure 1.**
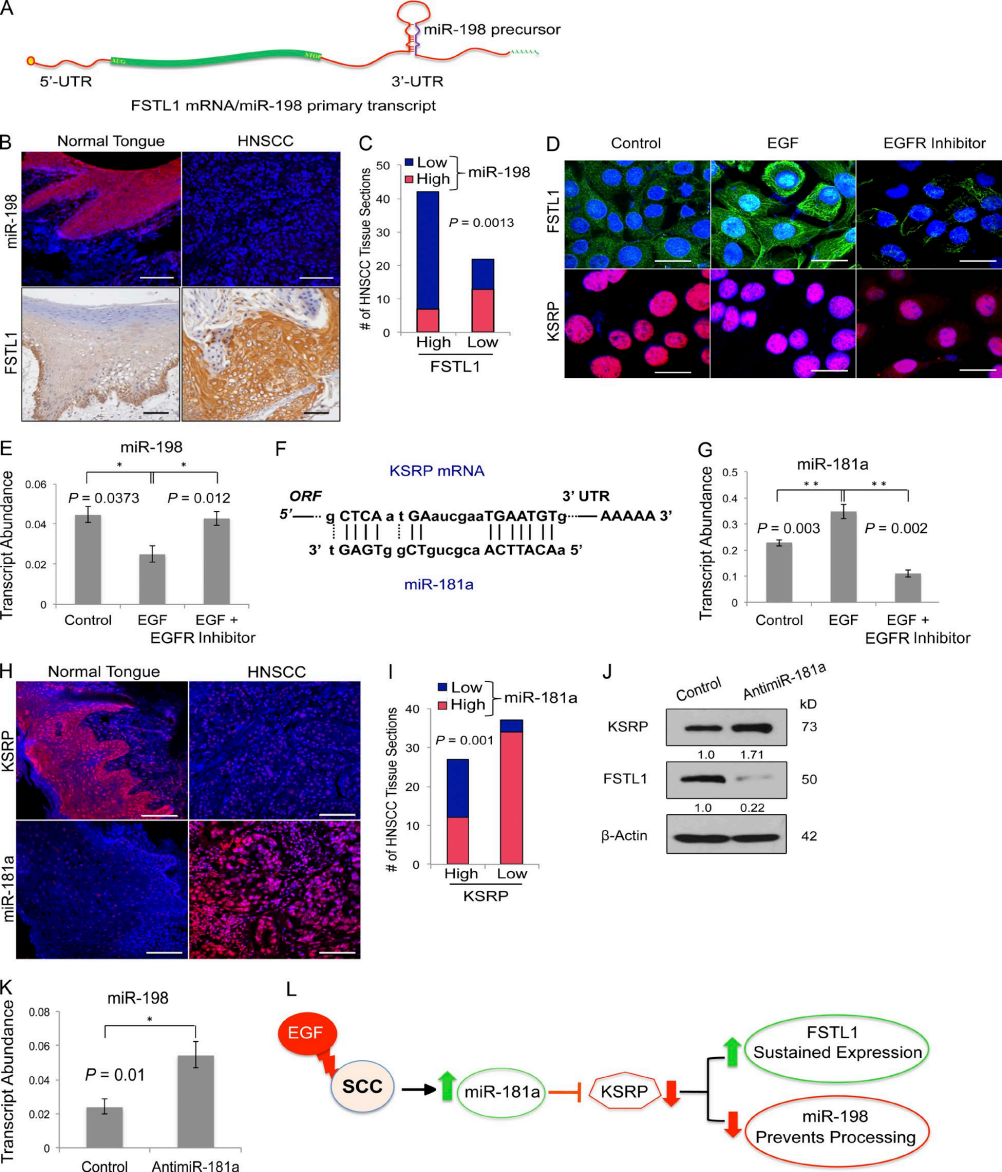
**EGF-driven microcircuitry hijacks the wound-healing switch.** (A) Schematic representation of the transcript that functions as either FSTL1 mRNA or primary miR-198 transcript. UTR, untranslated region. (B) Expression of miR-198 detected by in situ hybridization (red signal, top) and immunohistochemical localization of FSTL1 protein (brown stain, bottom) on normal tongue (*n* = 11) and HNSCC (*n* = 64) tissue sections. In both panels, nuclei are stained blue. Bars, 100 µm. (C) Quantification of FSTL1 and miR-198 expression in HNSCC sections. The plot shows an inverse correlation between FSTL1 and miR-198 expression in HNSCC sections. χ^2^ analysis with Bonferroni postcorrection was used to calculate p-values. (D) Immunocytochemistry staining using FSTL1 and KSRP specific antibodies on A253 cells treated with EGF and EGFR inhibitor (representative images of three independent experiments). Bars, 20 µm. (E) Histogram representing relative transcript abundance of miR-198 in A253 cells treated with EGF and EGFR inhibitor as measured by qRT-PCR. Error bar denotes SEM. Representative of three independent experiments. (F) miR-181a binding site in KSRP mRNA (NM_003685). (G) Histogram representing relative transcript abundance of miR-181a in A253 cells treated with EGF and EGFR inhibitor as measured by qRT-PCR. Error bar denotes SEM. Representative of three independent experiments. (H) Immunofluorescence staining of KSRP on normal tongue (*n* = 11) and HNSCC (*n* = 64) tissue sections (top). In situ hybridization with LNA probes specific for mature miR-181a on normal tongue (*n* = 11) and HNSCC (*n* = 64) tissue sections (bottom). Bars, 100 µm. (I) Quantification of KSRP and miR-181a expression in HNSCC sections. Plot shows an inverse correlation between KSRP and miR-181a expression in HNSCC sections. χ^2^ analysis with Bonferroni postcorrection was used to calculate p-values. (J) Western blot detection of KSRP, FSTL1, and β-actin on HNSCC cells transfected with control or anti–miR-181a (representative of three independent experiments). Band intensities were quantified by ImageJ and normalized to β-actin. (K) miR-198 relative transcript abundance in HNSCC cells treated with control oligonucleotides or anti–miR-181a. Error bars denote mean ± SEM. Student’s *t* test was used to calculate p-values. (L) Schematic representation of EGF-driven microcircuitry, which hijacks the molecular switch. *, P < 0.05; **, P < 0.01.

A bistable switch controls expression of two alternative products from a single transcript (Fig. 1 A). Epidermal wounding shuts off steady-state miR-198 expression in favor of FSTL1 translation, which enhances keratinocyte migration (Sundaram et al., 2013). To test our hypothesis that epithelial cell migration in progressive SCC results from dysregulation of the same switch, we examined miR-198 and FSTL1 expression in HNSCC. Fluorescent in situ hybridization revealed abundant miR-198 expression in normal tongue, which was absent in HNSCC tissue sections (Fig. 1 B). In those sections, elevated expression of FSTL1 relative to healthy sections was detected by immunohistochemistry (Fig. 1 B). We quantified miR-198 and FSTL1 expression across HNSCC sections and discovered an inverse correlation between the two; ∼60% of patients with increased expression of FSTL1 showed a concomitant decrease in miR-198 (Fig. 1 C and Fig. S1 F). Our observations that miR-198 is down-regulated in favor of FSTL1 expression support the involvement of a defective miR-198/FSTL1 switch in HNSCC.

Upstream regulation of the switch differs between epidermal wound healing and HNSCC. In skin, the trigger that down-regulates miR-198 and activates FSTL1 expression upon injury is TGF-β. Low levels of TGF-β in HNSCC (Fig. S1, A and F) suggest a different master regulator. Nevertheless, increased expression of EGF in HNSCC (Fig. S1, A and F) and down-regulation of miR-198 by EGF in breast cancer (Avraham et al., 2010) suggest a link between EGF and the molecular switch. To test this link, we stimulated HNSCC cells with exogenous EGF, which down-regulated miR-198 expression in favor of FSTL1. Ablating EGF signaling with an EGF receptor (EGFR) inhibitor (PD153035) eliminates this response (Fig. 1, D and E; and Fig. S1, B and C). Together, the data identify EGF as the upstream regulator of the defective switch in HNSCC.

miRNA-198 belongs to a small cohort of miRNAs that require KH-type splicing regulatory protein (KSRP) for processing (Trabucchi et al., 2009; Sundaram et al., 2013). To elucidate how EGF regulates the switch, we examined KSRP expression in HNSCC cells. KSRP is down-regulated upon EGF treatment and restored in the presence of PD153035 (Fig. 1 D and Fig. S1, B and C). These findings suggest a mechanism whereby EGF-mediated down-regulation of KSRP blocks miR-198 processing, permitting FSTL1 translation.

We postulated that EGF down-regulates KSRP via miR-181a. The KSRP 3′ untranslated region contains a binding site (Fig. 1 F) that is directly targeted by miR-181a. EGF treatment significantly increases miR-181a expression; this increase is counteracted by PD153035 (Fig. 1 G). Down-regulation of KSRP shows an inverse correlation with miR-181a expression pattern in HNSCC tissues (Fig. 1 H); ∼62% of patients showed increased miR-181a expression with a concomitant decrease in KSRP (Fig. 1 I and Fig. S1 F). Moreover, knockdown of miR-181a significantly increased KSRP expression, confirming that KSRP is a target of miR-181a (Fig. S1 D and Fig. 1 J). This led to a significant decrease in FSTL1 (Fig. 1 J) with a concomitant increase in miR-198 expression (Fig. 1 K), demonstrating the role of miR-181a as an upstream regulator of the molecular switch. In summary, the data decipher the upstream regulatory network of the defective switch in HNSCC, where EGF-mediated up-regulation of miR-181a down-regulates KSRP, leading to failure of miR-198 processing and up-regulation of FSTL1 (Fig. 1 L).

### The defective switch is proinvasive

**Figure 2.**
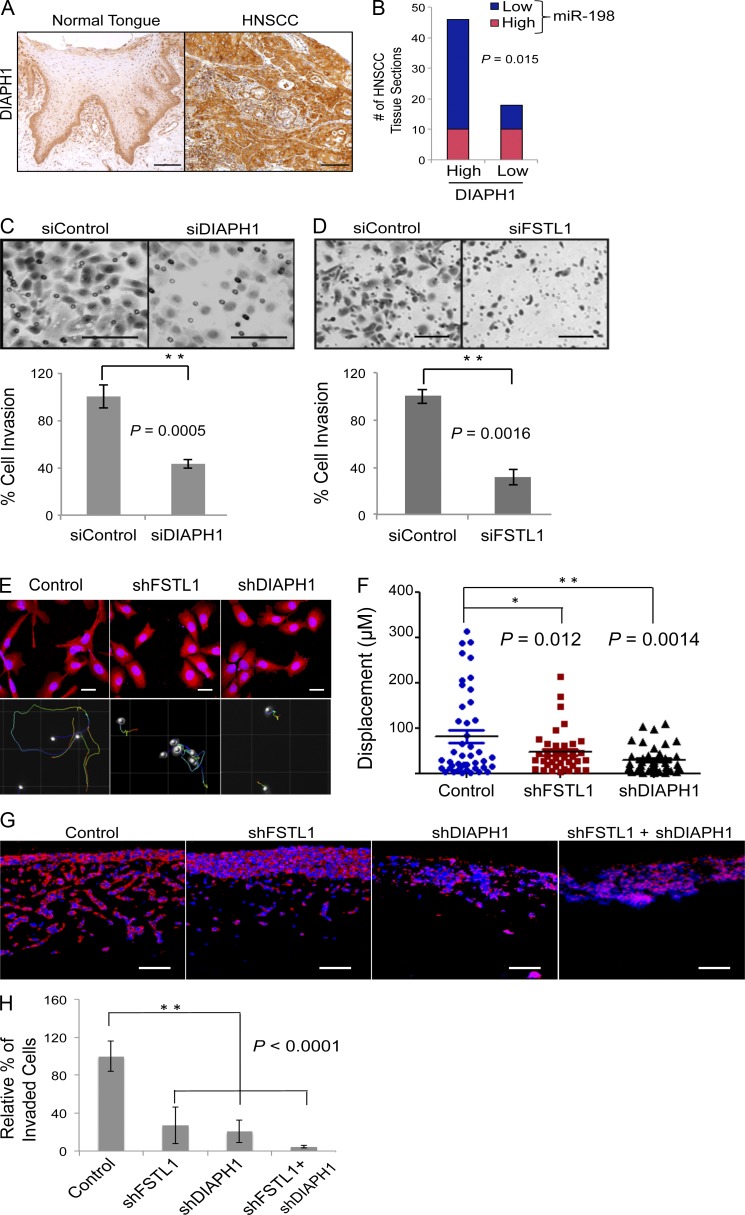
**The defective switch is proinvasive.** (A) Immunostaining of DIAPH1 on normal tongue (*n* = 11) and HNSCC (*n* = 64) tissue sections. In comparison with normal tongue, a significant increase in DIAPH1 expression is observed in HNSCC tissue sections. Bars, 100 µm. (B) Expression pattern of miR-198 and DIAPH1 across HNSCC tissue samples indicates a clear inverse correlation. (C and D) Boyden chamber invasion assay shows a significant decrease in the number of cells invading the chamber matrix in *siDIAPH1* or *siFSTL1* compared with control cells. Representative images of migrated cells detected with Giemsa staining (representative of five independent experiments). Error bars represent SD. Bars, 100 μm. (E) Morphology of SCC cells transduced with control shRNA or shRNA against FSTL1 or DIAPH1 (top) visualized using phalloidin-conjugated TRITC. Knockdown of FSTL1 or DIAPH1 showed a significant difference in morphology compared with control cells (top). Cell trajectory displacement over 24 h (bottom; *n* = 3). Bars, 10 μm. (F) Analysis of cell displacement in control, shFSTL1, and shDIAPH1 cells (representative of three independent experiments). (G) Organotypic invasion assay with shFSTL1 and shDIAPH1 independently or in combination or control SCC12 cells. SCC12 cells were detected by KRT14 staining (in red). Bars, 100 μm. (H) Histogram represents relative percentage of cells that invade the matrix (representative of three independent experiments; *, P < 0.05; **, P < 0.01). Error bars denote mean ± SEM.

Upon epidermal wounding, loss of miR-198 expression relieves repression on promigratory pathways that promote wound healing (Sundaram et al., 2013). DIAPH1 is among the derepressed miR-198 targets (Sundaram et al., 2013). A study showing that DIAPH1 depletion in colon cancer cells decreases metastatic potential (Lin et al., 2014) led us to investigate its role in HNSCC. Immunohistochemistry revealed up-regulation of DIAPH1 in HNSCC relative to healthy tongue (Fig. 2 A). miR-198 expression was found to correlate inversely with DIAPH1 expression (Fig. 2 B and Fig. S1 F); ∼80% of HNSCC patients showing increased DIAPH1 expression had reduced miR-198 expression. These data show that the defective switch promotes sustained expression of DIAPH1 and FSTL1 through its effects on miR-198 repression.

We evaluated the contribution of both genes to migration using Boyden chamber Transwell assays. Transient knockdown of either DIAPH1 or FSTL1 significantly reduced cell migration by 37.45 ± 3.65% and 34.23 ± 2.88%, respectively, compared with the control SCC12 cells (Fig. 2, C and D). Constitutive knockdown of either FSTL1 or DIAPH1 using specific shRNA (Fig. S1 E) resulted in a transition from mesenchymal to epithelial phenotype (Fig. 2 E). Using live cell imaging to track cellular trajectory over 24 h, we observed a significant decrease in cell migration with the loss of FSTL1 or DIAPH1 (Fig. 2, E and F). We also subjected shFSTL1, shDIAPH1, or combined knockdown cells to a three-dimensional organotypic assay that mimics in vivo stromal invasion (Nyström et al., 2005). Knockdown of either *FSTL1* or *DIAPH1* resulted in a considerable decrease in the number of invading cells compared with controls. Knocking down both genes virtually abolished cell invasion (Fig. 2, G and H). To analyze an effect caused by differences in cell proliferation, we injected shFSTL1 or shDIAPH1 cells individually or in combination subcutaneously in NSG mice. Bioluminescence imaging over a period of 4 wk did not show a significant defect in proliferation in vivo when we knocked down either FSTL1 or DIAPH1. When the two were knocked down in combination, we did see a reduction in cell growth; however, this difference was apparent only after 3 wk (Fig. S2 A). We also seeded cells in clonal densities and analyzed their proliferation rates. Proliferation was unaffected by knockdown of *FSTL1* or *DIAPH1*, independently or in combination (Fig. S2 B). Collectively, our findings strongly suggest that the switch defect is proinvasive; sustained expression of FSTL1 and DIAPH1 enhances migration and may facilitate metastasis.

### Prognostic significance of FSTL1-DIAPH1 gene pair

**Figure 3.**
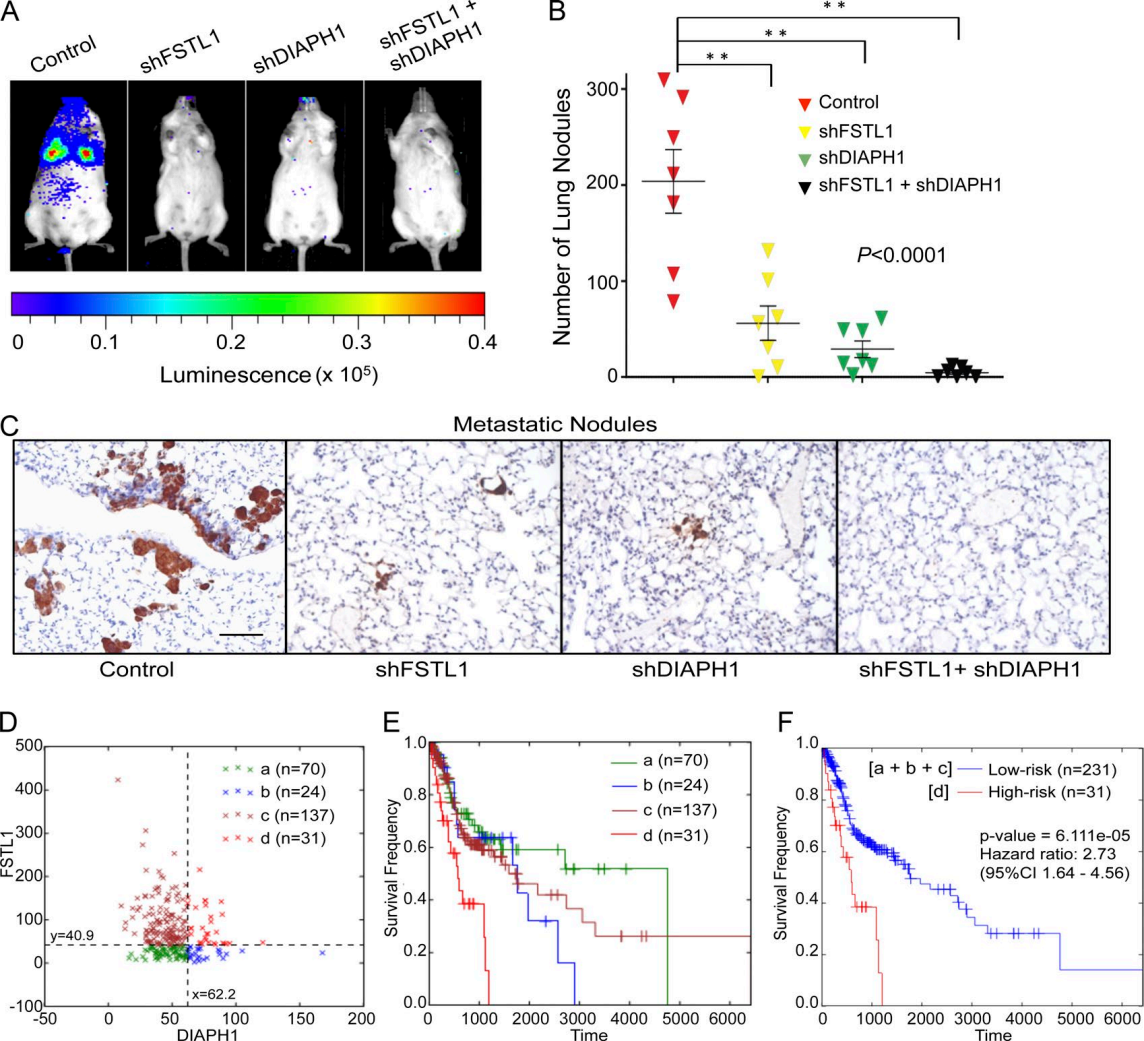
**Prognostic significance of FSTL1-DIAPH1 gene pair.** (A) Bioluminescence imaging of systemic metastasis by A253 cells expressing luciferase reporter and control shRNA or shRNA against FSTL1 or DIAPH1 individually or in combination (*n* = 7). (B) Representation of the total number of metastatic foci in lung sections. Error bars denote mean ± SEM. **, P < 0.0001. (C) Representative images of lung metastatic colonies detected by staining for KRT5 on day 26 postinjection. Bars, 100 μm. (D) Scatter plot of *FSTL1* and *DIAPH1* expression in 262 patients, classified into four distinct segments by 2D-DDg. (E) Kaplan–Meier survival curves of individual segments. (F) Kaplan–Meier survival curves with statistical significance by log-rank test of two groups, in which patients were grouped as low risk (segments a+b+c) and high risk (segment d).

To determine the in vivo role of FSTL1 and DIAPH1 in metastasis, luciferase-expressing A253 and FaDu cell lines were transduced with lentivirus encoding shFSTL1 or shDIAPH1 individually or in combination. Cells were implanted in immunodeficient NSG mice and evaluated for metastatic colonization. Silencing of FSTL1 or DIAPH1 individually or in combination reduced metastasis significantly compared with control (Fig. 3 A). Histological quantification revealed a substantial decrease in the total number of metastatic colonies (Fig. 3 B). Concurrent knockdown of FSTL1 and DIAPH1 virtually abolished lung colonization (Fig. 3, A–C; P < 0.0001). These results were consistent in both A253 and FaDu cell lines (Fig. S2, C–E) and present a strong case for the involvement of the FSTL1-DIAPH1 gene pair in metastatic colonization.

To understand the clinical significance of the defective switch, we interrogated the expression profile and clinical data of 262 head and neck cancer patients from the Cancer Genome Atlas (Cancer Genome Atlas Network, 2015). The survival significance of FSTL1 and DIAPH1 was simultaneously assessed via one-dimensional data-driven grouping (DDg). We discovered that both FSTL1 (borderline significant, log rank P = 0.0583) and DIAPH1 (log rank P = 0.00160) independently exhibited oncogenic behavior in which higher expression corresponded to a very modest overall patient survival. To study the combinatorial effect of FSTL1 and DIAPH1, we used two-dimensional (2D)-DDg (Motakis et al., 2009) to stratify the patient cohort into significant survival subgroups. Kaplan–Meier survival plots show that patients with low expression of both FSTL1 and DIAPH1 exhibit good overall survival (Fig. 3, D and E, green). In subgroups where either FSTL1 (Fig. 3, D and E, brown) or DIAPH1 (Fig. 3, D and E, blue) was highly expressed, survival prospects were lowered. Patients with high expression of both FSTL1 and DIAPH1 (Fig. 3, D and E, red) had the lowest overall survival rates. Our results indicate that FSTL1 and DIAPH1 individually exhibit oncogenic behavior. When combined, the prognostic features of the gene pair generate robust patient stratification (Fig. 3 F), suggesting a combinatorial effect between the genes in the prognosis of HNSCC.

### EGF-driven microcircuitry steers a two-pronged pathway towards metastasis

**Figure 4.**
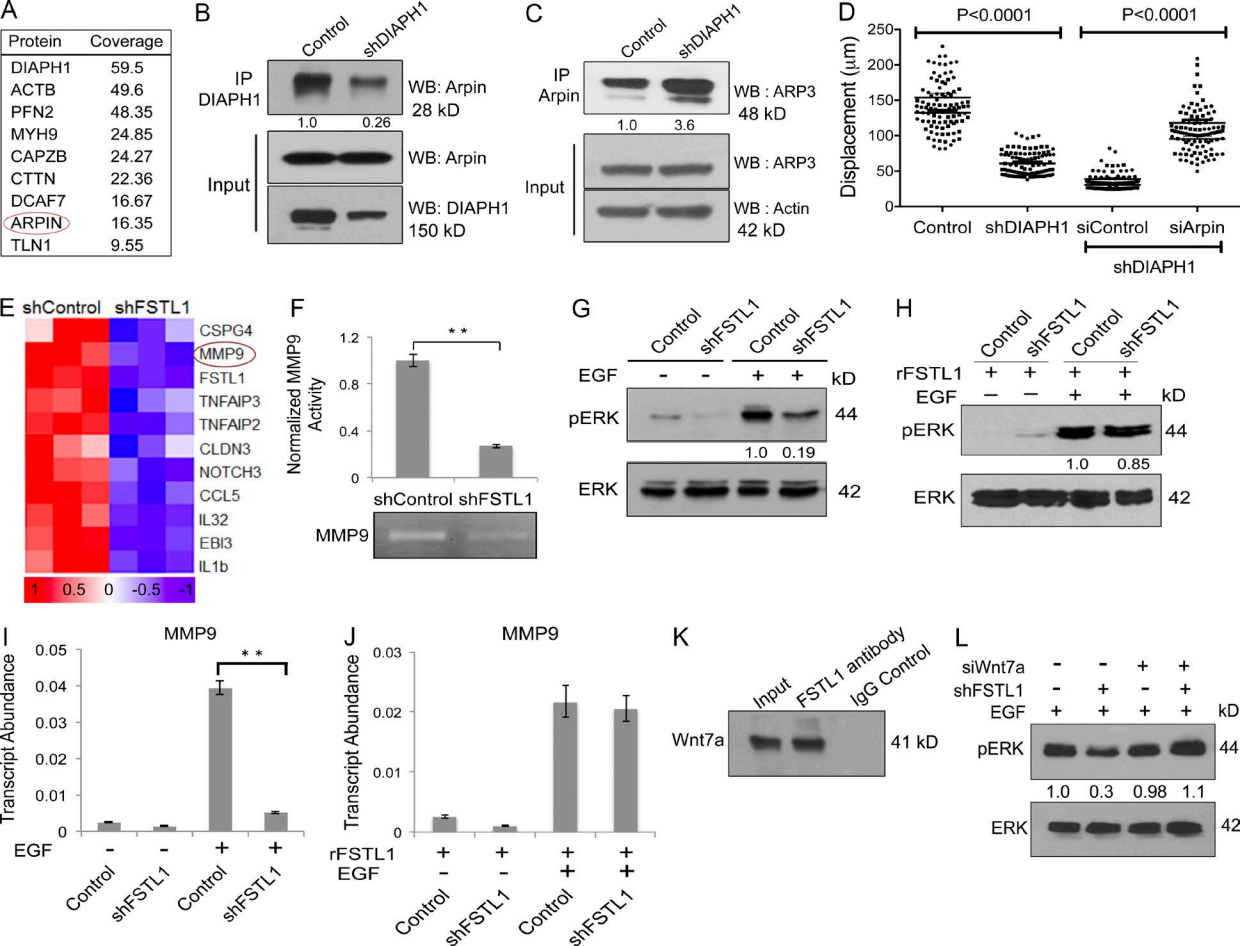
**EGF-driven microcircuitry steers a two-pronged pathway toward metastasis.** (A) List of selected interacting partners of DIAPH1 shortlisted from BioID. (B) Western blot analysis on SCC12 lysates immunoprecipitated with anti-DIAPH1 and probed for Arpin (top). Input cell lysates were probed for Arpin (middle) and DIAPH1 (bottom). Representative image is from three independent experiments. (C) Western blot on SCC12 control or shDIAPH1 cell lysates immunoprecipitated with anti-Arpin and probed for Arp3 (top). Input cell lysates were probed for Arp3 (middle) and β-actin (bottom). Representative image is from three independent experiments. IP, immunoprecipitation; WB, Western blotting. (D) Analysis of cell displacement upon DIAPH1 knockdown and rescue with concurrent knockdown of both DIAPH1 and Arpin. Representative image is from three independent experiments. (E) Heat map showing expression values of selected genes from microarray data of SCC12 cells transfected with control shRNA or shFSTL1. Expression values displayed in shades of red (high) or blue (low) relative to the individual mean value of the gene in linear scale. (F) Gelatin zymography and quantification of band intensities represented as a histogram (top). **, P < 0.001; error bars represent SD (*n* = 3). (G) Western blot on control or shFSTL1 SCC cell lysates from EGF-pretreated cells. Lysates were probed for phosphoERK or total ERK. (H) Western blot showing rescue of phosphoERK upon treatment with recombinant FSTL1 (*n* = 2). (I) Relative transcript abundance of MMP9 in control or shFSTL1 A253 cells treated with EGF. **, P < 0.001. Error bars represent SD (*n* = 5). (J) Rescue of MMP9 expression upon treatment with recombinant FSTL1. Error bars represent SD (*n* = 3). (K) A253 cell lysates were immunoprecipitated with IgG or anti-FSTL1 antibody and probed for Wnt7a by Western blot analysis. Representative image is from three independent experiments. (L) Western blot on lysates from shFSTL1 cells transfected with specific siRNA against Wnt7a and cells treated with EGF posttransfection. Lysates were probed for phosphoERK or total ERK (*n* = 3).

To identify interacting partners of DIAPH1 relevant to its role in metastasis, we performed proximity-dependent biotin identification (BioID) analysis (Roux et al., 2013). BioID identified Arpin as a candidate DIAPH1 partner (Fig. 4 A and Fig. S3 A). Arpin, a competitive inhibitor of the Arp2/3 complex, contributes to lamellipodia collapse and restricts cell migration (Dang et al., 2013). This function is opposite to that of DIAPH1, which cooperates with Arp2/3 to drive lamellipodia formation and cell migration (Isogai et al., 2015). We hypothesize that sequestration of Arpin by DIAPH1 prevents its association with Arp2/3, thereby enhancing cell migration.

To validate the DIAPH1–Arpin interaction, cellular lysates from control or shDIAPH1 HNSCC cells were subjected to immunoprecipitation with anti-DIAPH1 antibody and analyzed by Western blot. Arpin was significantly enriched in controls relative to DIAPH1-depleted samples, confirming its interaction with DIAPH1 (Fig. 4 B and Fig. S3, B and C). Similar immunoprecipitation was performed using an anti-Arpin antibody, with immunoprecipitates probed for Arp3. Arp3 was significantly enriched in DIAPH1-depleted samples compared with controls, suggesting that DIAPH1 sequestration of Arpin blocks its interaction with Arp3 (Fig. 4 C and Fig. S3, D and E). These results were consistent across multiple cell lines. Concurrent knockdown of Arpin (Fig. S3 F) rescued the migration defect caused by DIAPH1 knockdown (Fig. 4 D). Together, the data demonstrate that DIAPH1 sequesters Arpin, which blocks directional persistence of migration and alleviates its repression of Arp2/3 activity, promoting cell migration and thus contributing to metastasis.

Having elucidated the regulatory network downstream of miR-198 and its derepressed target DIAPH1 in HNSCC, we proceeded to characterize the contribution of FSTL1 to metastasis. Using microarrays, we compared gene expression between control and shFSTL1 SCC12 cells. Knockdown of FSTL1 resulted in significant down-regulation of MMP9 relative to controls (Fig. 4 E). Microarray data were validated by quantitative RT-PCR (qRT-PCR; Fig. S3 G). Moreover, a decrease in MMP9 activity upon knockdown of FSTL1, relative to control (Fig. 4 F), suggests that FSTL1 is an upstream regulator of MMP9 expression.

MMP9 expression is known to be stimulated via EGF-mediated ERK pathway activation (McCawley et al., 1999; O-charoenrat et al., 2000; Zuo et al., 2011). We investigated activation of ERK in HNSCC cells by probing EGF-treated, shFSTL1, or control cell lysates for phosphoERK. FSTL1 knockdown reduced EGF-mediated ERK phosphorylation (Fig. 4 G), but addition of recombinant FSTL1 rescued ERK phosphorylation (Fig. 4 H). qRT-PCR analysis confirmed that EGF-mediated MMP9 up-regulation fails in the absence of FSTL1 (Fig. 4 I and Fig. S3 H). Nevertheless, adding recombinant FSTL1 rescued MMP9 expression (Fig. 4 J). Our data not only substantiate the role of EGF in regulating the expression of MMP9 (Ruokolainen et al., 2004; O’Grady et al., 2007), but also emphasize the role of FSTL1 as an upstream regulator of MMP9.

**Figure 5.**
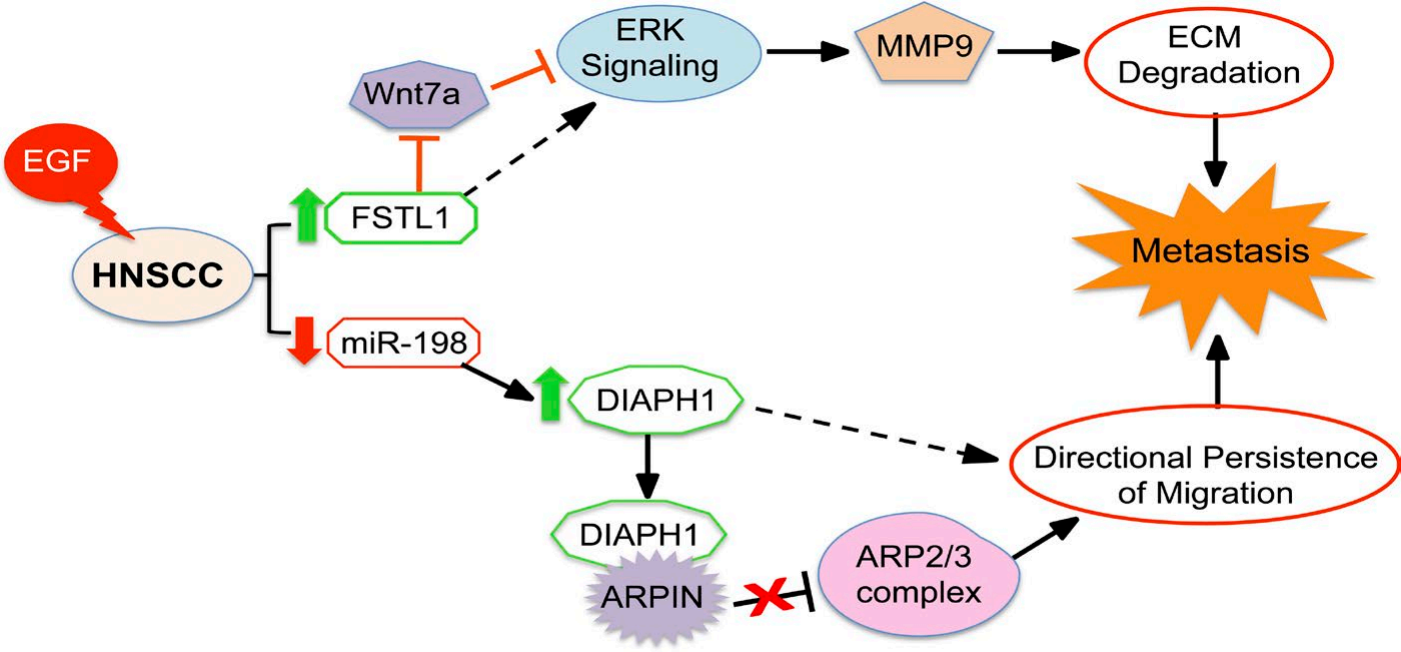
**Schematic representation of the two-pronged pathway toward metastasis.** Model depicting hijacking of miR-198/FSTL1 molecular switch by EGF, on a dual road to metastasis.

To understand how FSTL1 regulates ERK phosphorylation, we performed analysis with Biological General Repository for Interaction Datasets (BioGRID; Chatr-aryamontri et al., 2015). This analysis revealed multiple interacting partners of FSTL1, including Wnt4, Wnt5a, Wnt7a, and Wnt10b (Fig. S3 I). HNSCC cell lysates were immunoprecipitated with anti-FSTL1 antibody or IgG control and probed for these Wnts. Western blot results indicated that only Wnt7a specifically interacts with FSTL1 in HNSCC (Fig. 4 K). Because Wnt7a has effects opposite those of FSTL1, inhibiting EGF signaling (Winn et al., 2005) and suppressing tumor cell invasion (Winn et al., 2006; Ramos-Solano et al., 2015), we suggest that FSTL1 sequesters and blocks Wnt7a, thus enabling EGF-mediated ERK phosphorylation. To test this hypothesis, we performed siRNA knockdown of Wnt7a in shFSTL1 cells. After siRNA transfection, cells were treated with EGF to stimulate ERK phosphorylation. Knocking down Wnt7a (Fig. S3 J) rescued the inhibition of ERK phosphorylation caused by FSTL1 knockdown (Fig. 4 L). This suggests that Wnt7a inhibits EGF-mediated ERK phosphorylation. Together, these results describe a model whereby FSTL1 interacts with Wnt7a, blocking Wnt7a-mediated repression of ERK phosphorylation in HNSCC. ERK phosphorylation stimulates MMP9 expression, leading to ECM degradation, which promotes metastasis in HNSCC (Fig. 5).

### Concluding remarks

Wound healing is a self-limiting dynamic event. After wound closure, activated keratinocytes revert to a quiescent state and redifferentiate to restore the epidermal barrier (Freedberg et al., 2001). In carcinoma, keratinocytes maintain a promigratory gene expression profile and remain activated.

FSTL1 expression must be tightly regulated such that it can perform its intended physiological function while avoiding the detrimental consequences of sustained inappropriate expression. The miR-198/FSTL1 switch is critical to this regulation. Here we demonstrate the hijacking of the molecular switch in HNSCC. miR-198 expression is lost, allowing persistent expression of promigratory FSTL1 and DIAPH1 (Fig. 5). Although normal wound-healing events are insufficient to trigger tumor formation, aberrant maintenance of the wound microenvironment promotes increased cell migration, leading to metastasis (Troester et al., 2009; Shalapour and Karin, 2015). We propose that the defective switch may represent a target for development of innovative therapeutic strategies to treat HNSCC with improved patient outcome.

## Materials and methods

### Cell culture and SCC tissue array

HNSCC cell lines (A253, SCC12, and FaDu) were cultured in RM^+^ media (3:1 DMEM and F12 Ham’s medium supplemented with 10% FBS, 0.5 µg/ml hydrocortisone, 5 µg/ml insulin, 5 µg/ml transferrin, 13 ng/ml liothyronine, 1% glutamine, 10 ng/ml EGF, and penicillin/streptomycin). HNSCC tissue arrays (HN803b) were obtained from US Biomax. Five HNSCC sections were negative for KRT14/KRT5 and therefore omitted for further study. Human foreskin dermal fibroblasts and lenti-X 293T (Clontech) were cultured in DMEM supplemented with 10% FCS and penicillin/streptomycin.

### Antibodies and proteins

Antibodies used in this study are as follows. Goat anti-FSTL1 (ab11805; Abcam), rabbit anti-FSTL1 (Proteintech), rabbit anti-DIAPH1 (5486; Cell Signaling Technology), rabbit anti-KSRP (A302-22A; Bethyl Laboratories), mouse anti-KRT14 (clone LLO01), mouse anti-EGF (clone 10825; R&D Systems), mouse anti-TGFβ1 (Novacastra), rabbit anti-GFP (Covance), rabbit anti-cMyc (Sigma-Aldrich), and rabbit anti-Wnt7a (ab100792; Abcam). Streptavidin Alexa Fluor 555, chicken anti-goat Alexa Fluor 488, and donkey anti-rabbit/mouse Alexa Fluor 488/555 were from Molecular Probes. Rabbit anti-Arp3 (4738), phospho (9101), and total ERK antibodies (9102) were from Cell Signaling Technology. Rabbit anti-Arpin (ABT251) was purchased from Merck Millipore. Recombinant human FSTL1 (His-tagged) expressed and purified from mammalian cells was purchased from Thermo Fisher Scientific.

### Stable knockdown and microarray analysis

shRNAs were designed against the open reading frame of FSTL1/DIAPH1 sequences using siRNA wizard software (InvivoGen). shRNAs were cloned in lentiviral shRNA expression vector in which H1 promoter drives the expression of shRNA by RNA polymerase III. Third-generation lentiviruses were produced in Lenti-X 293T cells (Clontech) by cotransfection of shControl/shFSTL1/shDIAPH1 vectors and lentiviral packaging plasmid mix (pMDLg/pRRE, pRSV-Rev, and pMD2.G). HNSCC cell lines were transduced with either control lentivirus encoding a scrambled shRNA or shRNA against FSTL1/DIAPH1. Cells with stable integration of lentiviruses were selected with 2 µg puromycin for 2 wk. Total RNA was isolated from cells using the Exiqon miRcury RNA isolation kit. 250 ng total RNA was converted into biotinylated complementary RNA using a TargetAmp Nano-g Biotin-aRNA labeling kit (Epicenter). 750 ng biotinylated cRNA was hybridized to HT-12 V4 expression bead chip (Illumina) using samples in triplicate. Hybridization, washing, and scanning were performed according to the manufacturer’s protocol. Data extracted was normalized and analyzed using Illumina BeadStudio. Microarray data has been deposited in Gene Expression Omnibus database, accession no. GSE 63329.

### Boyden chamber invasion assays

Transient knockdown of FSTL1 or DIAPH1 was achieved by transfection of 50 nM On-target Plus Smart-pool siRNA (Dharmacon; Thermo Fisher Scientific) using RNAimax transfection reagent as per manufacturer’s protocol. A nontargeting siRNA was transfected as negative control. In vitro invasion assays were performed in BD biocoat Matrigel invasion assay chambers. 2 d after transfection, cells were harvested and seeded onto the upper chamber in RM^+^ medium without serum. Complete RM^+^ medium was used as a chemoattractant in the lower chambers. 20 h after seeding, cells above the membrane were wiped out with a cotton swab, and the invaded cells were fixed in methanol and stained with Giemsa solution. Cell invasion was expressed as the percentage of cells invaded in six microscopic fields per chamber in five biological replicates.

### Organotypic invasion assay and quantification

Organotypic assays were set up as described previously (Gaggioli et al., 2007). In brief, in ice-cold conditions, equal amounts of Matrigel (354234; BD Biosciences) and rat tail collagen type I (354236; BD Biosciences) with 1-10th volume of FCS and 10× DMEM were mixed and adjusted to neutral pH by addition of 0.1 N NaOH. Approximately 800 µl of this solution was added to Millicell hanging cell culture inserts and placed in 12-well plates (Nunc) for 1 h at 37°C for solidification. Complete fibroblast medium was added to both top and bottom of the insert and left overnight at 37°C. Human dermal fibroblasts (1 × 10^5^) were mixed with HNSCC cell line (2.5 × 10^5^), seeded on the gel, and allowed to grow submerged in RM**^+^** medium for 24 h. Inserts were lifted to air–liquid interface the next day, and cultures were maintained in RM**^−^** medium (without EGF) for 2 wk, after which the organotypic gels were processed. The depth of invasion and total number of Keratin 14–positive cells were quantified using ImageJ software.

### Immunohistochemistry

5-µm tissue sections were mounted on polylysine-coated glass slides (Thermo Fisher Scientific). Sections were washed in xylene and rehydrated using decreasing ethanol concentrations and finally in PBS. Endogenous peroxidase activity was quenched by immersing the slides in 3% hydrogen peroxide for 30 min. Antigen retrieval was performed using a programmable pressure cooker with target retrieval solution, pH 6.0 (Dako). Nonspecific reactivity in the tissues was blocked by incubation in 10% goat serum in PBS before incubating with the primary antibody at room temperature. Unbound primary antibodies were removed before incubation with species-matched secondary HRP-labeled polymer antibodies (Dako). Chromogen 3,3′-diaminobenzidine (Dako) was used as substrate for color development. Slides were counterstained with hematoxylin before dehydration and mounted with DPX (Sigma). For fluorescent immunodetection, species-specific secondary antibodies conjugated to Alexa Fluor 488/555 were used instead of HRP-labeled polymer antibodies. Sections were washed, counterstained with DAPI (100 ng/ml), and mounted using FluorSave (Calbiochem). For experiments in which goat primary antibodies were used, 5% BSA in PBS was substituted for 10% goat serum. Images were acquired on a Zeiss Axioimager microscope (for bright-field imaging) or an Olympus FluoView FV1000 (for fluorescent antibody detection).

### miRNA in situ hybridization

5-µm sections were processed and boiled in pretreatment solution (Panomics) and washed in PBS, followed by protease (Panomics) treatment at 37°C. Sections were incubated with LNA probes (5′-DIG labeled LNA probes specific for miR-198/miR-181a or scrambled probe with no homology to known vertebrate miRNAs; Exiqon) in hybridization buffer (Roche) at 51°C for 4 h. After stringent washes with 5×, 1×, and 0.3× SSC buffer, sections were blocked with 10% goat serum and further incubated with anti-DIG alkaline phosphatase (Roche) overnight at 4°C. Sections were washed in PBS-T (0.1%), and miRNA-bound LNA probes were detected by Fast Red substrate (Panomics). After counterstaining with DAPI, slides were mounted using FluorSave (Merck). Image acquisition was performed with an Olympus FluoView FV1000 with TRITC filter.

### In vivo lung metastasis and subcutaneous tumorigenic assays

6- to 8-wk-old female NOD-*scid* IL2Rg^null^ inbred mice were obtained from the Jackson Laboratory and housed in a specific pathogen–free animal facility. The animals were fed with irradiated mouse diet and autoclaved reverse osmosis–treated water. All animal procedures were performed in accordance with a protocol approved by the Institutional Animal Care and Usage Committee (IACUC 120793). A253 or FaDu cells constitutively expressing luciferase, transduced with lentiviruses expressing control shRNA or shRNAs against FSTL1, DIAPH1, or both in combination, were harvested with trypsin/EDTA. Cells were washed and resuspended at a concentration of 5 × 10^6^ cells/ml. A total of 0.5 × 10^6^ cells were injected into mice via tail vein, and cohorts of seven mice were used in each group. Survival and successful injection of the cells was monitored by detection of bioluminescence in the lungs after 24 h using IVIS Spectrum In Vivo Imaging System (Xenogen; PerkinElmer). In each imaging session, a total of 150 mg Luciferin per kg body weight was administered into the peritoneal cavity. Mice were imaged 9 min after Luciferin injection to ensure consistent photon flux. The bioluminescent signal was expressed in photons per second and displayed as an intensity map. The image display was adjusted to provide optimal contrast and resolution in the image without affecting quantitation. After acquisition, all images were normalized to units of mean efficiency, displayed in the same scale of luminescence intensity, and analyzed using the Living Image 4 software (Xenogen; PerkinElmer). Luminescence from the cells was measured in the lungs using a region of interest tool. 30 d after injection, mice were killed and lungs were harvested, fixed, and processed into formalin-fixed, paraffin-embedded blocks. Cancer cells in micrometastatic foci were detected by immunohistochemistry using a rabbit anti–human KRT5 antibody. To assess the survival of HNSCC cells in vivo, 5 million cells were injected along with Matrigel in flank regions of mice, and bioluminescence was measured.

### Zymography

20 µl cell culture supernatant was loaded and resolved on to SDS-PAGE containing 0.15% gelatin followed by two washes in 2.5% Triton X-100. Gel was soaked in a developer buffer containing 50 mM Tris HCl, pH 7.4, 150 mM NaCl, and 10 mM CaCl_2_ overnight at 37°C and visualized by staining the gel in 0.2% Coomassie Brilliant Blue R-250 for 1 h, followed by multiple destaining rounds in 30% methanol and 7% acetic acid. Band intensity was quantified by ImageJ and plotted after normalizing the values to background intensity. The position of MMP9 was identified by comigration of recombinant MMP9 in the same gel.

### Biotin-streptavidin affinity purification of DIAPH1 interacting partners

Full-length cDNA for DIAPH1 (accession no. NM_005219) was purchased from Origene. DIAPH1 open reading frame was PCR amplified and cloned as a fusion protein N-terminally with a myc-tagged BirA in pTRIPZ vector system. The entire myc-BirA-DIAPH1 cassette is under the control of tetracycline-inducible promoter system (Tet-ON) for the stringent control of fusion protein expression. For affinity purification of biotinylated interacting partners of DIAPH1, 293T cells were transfected with 20 µg plasmid DNA using calcium phosphate transfection. Cells were selected with puromycin (2 µg/ml), and 72 h after transfection, cells were supplemented with doxycycline (Dox; 5 µg/ml) for 24 h to induce transgene expression. To induce biotinylation of interacting partners, 50 µM biotin was added to cells and incubated for 24 h. Cells were harvested by trypsinization and subjected to biotin affinity capture purification (BioID; Roux et al., 2013). In brief, cells were lysed in buffer containing 50 mM Tris Cl (pH 7.4), 500 mM NaCl, and 0.2% SDS with 1× protease inhibitor cocktail (Roche). After clarifying the cell debris by centrifugation, lysates were incubated with Dynabeads (MyOne Streptavidine C1; Life Technologies) overnight. Unbound and nonspecific proteins were removed after wash buffer formulation as described previously (Roux et al., 2013). After a final wash with 50 mM Tris Cl, pH 7.4, proteins bound to beads were subjected to mass spectrometry analysis (see next section). For initial validation of BioID methodology, cells were transfected in coverslips. The fusion protein and biotinylated proteins were detected by rabbit anti-myc and streptavidin-conjugated Alexa Fluor 555 (Invitrogen), respectively.

### Mass spectrometry and data analysis

For mass spectrometric analysis, proteins bound to streptavidin beads were digested on-bead according to the protocol of Xie et al. (2016). The peptides were injected and separated using an Orbitrap Fusion Tribrid mass spectrometer (Thermo Fisher Scientific) and coupled to proxeon EASY-nLC 1000 liquid chromatography (LC-MS/MS) in a 120-min gradient. Raw data were analyzed using Proteome Discoverer (v1.4.0.288; Thermo Fisher Scientific) and Scaffold 4.1 (Proteome Software) against Human Uniprot database with the following parameters: precursor mass tolerance (MS) 25 ppm, MS/MS 0.6 Da, three miss cleavages; static modifications of carboamidomethyl, variable modifications oxidation (M), biotin (K), and deamindation (NQ; Searle, 2010). For protein identification, we conducted a high-confidence database search with peptide target false discovery rate (strict) of 0.01% and target false discovery rate (relaxed) of 0.05% based on q values. Identified peptides were grouped into individual protein cluster by Scaffold. The list of proteins obtained by this method was further shortlisted (Kim et al., 2014). Proteins identified in cells with no Dox treatment but with biotin, proteins commonly found in BioID methodology, and proteins with less than 8% peptide coverage (Kim et al., 2014) were subtracted from the list. The raw mass spectrometry datasets and curated DIAPH1 interacting partners have been submitted to the international standard data repository for proteomes (jPOSTrepo; Okuda et al., 2017) under accession no. JPST000273.

### Coimmunoprecipitation

Total cell extract was prepared from HNSCC cells by directly lysing the cells in RIPA buffer in culture wells. Cells were lysed at five pulses of sonication followed by clarification at 16,000 RCF for 10 min at 4°C. Coimmunoprecipitation analysis for selected proteins was performed using a kit (Pierce) per the manufacturer’s recommendations. Proteins bound to beads were eluted in Laemmli buffer, resolved through 10% SDS-PAGE, and subjected to Western blot analysis.

### Live cell imaging

10 × 10^3^ cells were seeded in a glass-bottom culture dish (Ibidi) for 24 h. A drop of Nucblue live cell stain (R37605; Life Technologies) was added to the cells in the culture medium and incubated for 30 min. The cells were washed with PBS, and fresh medium was added. The live cell migration was followed for 24 h, using Olympus IX-83 LCI RM 5.17 live cell imaging system. The data were processed and analyzed using Imaris 8.1.2 software tool. For rescue experiments, SCC12 and FaDu shDIAPH1 cells were transiently transfected with 50 nM of a control nontargeting siRNA pool or specific siRNAs for Arpin. 2 d after transfection, cells were subjected to live cell imaging to assess the effect of siArpin on cell migration.

### Tissue microarray quantification

Images were acquired with a 10× objective to cover the entire tumor section. Blinded quantification of staining intensity on tissue array was performed by two independent observers, and intensity was classified as negative, weak, moderate, and strong by taking a mean of independent observations as described in Schmit et al. (2009). For correlation analysis between paired genes (FSTL1 vs. miR-198, KSRP vs. miR-181a, and DIAPH1 vs. miR-198), sections with negative/weak staining were considered as low, and moderate/strong staining intensities were considered as high. Statistical testing was performed with χ^2^ analysis with Bonferroni postcorrection. p-values <0.05 were considered significant (Chao et al., 2014).

### Cell proliferation and viability assay

To assess the effect of FSTL1/DIAPH1 knockdown on cell proliferation, HNSCC cells with shFSTL1, shDIAPH1, or both were seeded at a density of 500 cells/well in 96 wells in complete medium. Cell proliferation was measured at different days after seeding using CellTiter-Glo Luminescent Cell viability assay per manufacturer’s instructions. Experiments were performed in two biological replicates with at least eight technical replicates per condition.

### Western blotting

Cells were directly lysed from six-well plates by scraping in RIPA buffer. After clarifying the lysate by centrifuging at 13,000 rpm at 4°C, total protein was quantitated by Bradford Protein Assay (Bio-Rad). Equal amounts (30 µg) of total protein were subjected to SDS-PAGE followed by Western blotting with standard protocols. After primary and secondary antibody incubation and washing, proteins were visualized by ECL Western detection reagent (Millipore Crescendo). Band intensities were quantified by ImageJ. Signal intensities were normalized to their appropriate loading controls (actin or total ERK).

### Cell treatments

Cells were seeded on six-well plates in complete medium. 24 h after seeding, cells were further starved of serum in DMEM containing 0.1% FBS. For Fig. 1 (D, E, and G) and Fig. S1 (B and C), cells were treated with either EGF alone (10 ng/ml) or EGF and PD153035 (0.5 mM) for 24 h. For the detection of phosphoERK after serum starvation, cells were treated with EGF (10 ng/ml) for 20 min. In rescue experiments, cells were pretreated with recombinant FSTL1 (50 ng/ml) in serum-free media before the addition of EGF.

### DDg method

One-dimensional DDg is a computational and statistical approach to identify an optimal expression cutoff on a linear scale that provides maximal and most statistically significant stratification of the survival curves (Motakis et al., 2009). 2D-DDg further extends the idea of DDg and evaluates the potential synergistic effect of gene pairs in patient prognosis. In brief, 2D-DDg identifies one expression cutoff on each orthogonal axis (each representing one gene of the gene pair) that collectively could stratify patients into two subgroups with the most significantly different survival curves. The methods of DDg and 2D-DDg were previously used to identify and experimentally validate molecular signatures of ovarian cancer (Tang et al., 2014) and breast cancer (Grinchuk et al., 2015) as well as to evaluate the survival significance of gene features in glioblastoma (Chan et al., 2012).

### Statistical analysis

Values are reported as the mean ± standard error. Statistical significance between two samples was determined with two-tailed Student’s *t* test or one-way analysis of variance when comparing multiple groups.

### Online supplemental materials

Fig. S1 shows the absence of TGF-β ligand in HNSCC sections and effect of EGF/EGFR inhibitor on miR-198/FSTL1 molecular switch and its regulators; tables in Fig. S1 shows histological quantification of the components and regulators of the molecular switch in HNSCC tissue microarray and normal tongue sections. Fig. S2 shows additional in vivo metastasis data in FaDu, an HNSCC cell line. Fig. S3 shows BioID validation by immunocytochemistry, validation of DIAPH1-Arpin interaction in two additional HNSCC cell lines, Arpin knockdown, qRT-PCR data of differentially expressed candidates from microarray, and BioGRID analysis depicting FSTL1 interacting partners.

## Supplementary Material

Supplemental Materials (PDF)
