# Supplementary material for: EGF hijacks miR-198/FSTL1 wound-healing switch and steers a two-pronged pathway toward metastasis
Source: J Exp Med. 2017 Oct 2;214(10):2889–900. doi: 10.1084/jem.20170354 (PMC5626400; doi:10.1084/jem.20170354)
Supplement: Supplemental Materials (PDF) [file JEM_20170354_sm.pdf]

SUPPLEMENTAL MATERIAL

Sundaram et al., https://doi.org/10.1084/jem.20170354

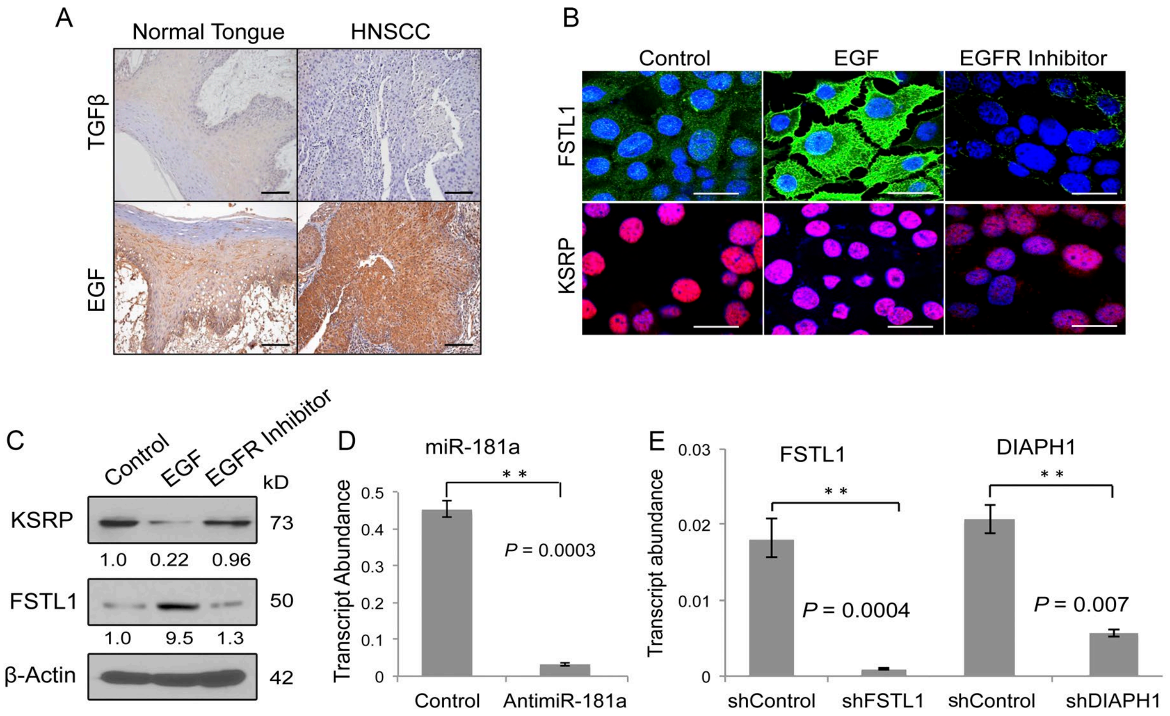

F

Cancer tissues							
Expression grade	miR-198	FSTL1	DIAPH1	miR181a	KSRP	TGF-β	EGF
Negative (-)	23/64 (36%)	1/64 (1.5%)	0/64 (0%)	0/64 (0%)	5/64 (8%)	30/64 (47%)	4/64 (6%)
Weak (+)	21/64 (33%)	21/64 (33%)	8/64 (12%)	18/64 (23%)	32/64 (50%)	31/64 (48%)	26/64 (41%)
Moderate (++)	15/64 (24%)	26/64 (40%)	38/64 (60%)	21/64 (33%)	22/64 (34%)	3/64 (5%)	28/64 (44%)
Strong (+++)	5/64 (8%)	16/64 (25%)	18/64 (28%)	25/64 (44%)	5/64 (8%)	0/64 (0%)	6/64 (9%)
Cancer adjacent and normal tongue tissues							
Expression grade	miR-198	FSTL1	DIAPH1	miR181a	KSRP	TGF-β	EGF
Negative (-)	0/11 (0%)	2/11 (18%)	0/11 (0%)	1/11 (9%)	1/11 (9%)	5/11 (45%)	2/11 (18%)
Weak (+)	2/11 (18%)	8/11 (73%)	7/11 (64%)	10/11 (91%)	1/11 (9%)	6/11 (55%)	8/11 (73%)
Moderate (++)	4/11 (36%)	1/11 (9%)	4/11 (36%)	0	7/11 (64%)	0	1/11 (9%)
Strong (+++)	5/11 (46%)	0/11 (0%)	0/11 (0%)	0	2/11 (18%)	0	0/11 (0%)
Chi-square P value between normal and cancer	P=0.0017	P=0.0013	P= 0.0546	P=.0001	P=0.08	N.S	P= 0.05

Figure S1. **EGF is an upstream regulator of miR-198/FSTL1 switch.** (A) Detection of TGF-β1 ligand (top) and EGF ligand (bottom) by immunohistochemistry in normal tongue (*n* = 11) and HNSCC (*n* = 64) tissue sections. Bar, 100 µm. (B) Immunocytochemistry staining using FSTL1 and KSRP specific antibody on FaDu cells treated with EGF. Bar, 20 µm. Images are representative of four independent experiments. (C) Western blot detection of KSRP, FSTL1, and β-actin from SCC12 cell lysates treated with EGF or EGFR inhibitor. Images are representative of two independent experiments. (D) Histogram representing miR-181a relative transcript abundance in SCC12 cells treated with control or anti–miR-181a oligonucleotides (*n* = 3). (E) Histogram representing *FSTL1* or *DIAPH1* transcript abundance in SCC12 cells transduced with control shRNA or shRNA against *FSTL1* or *DIAPH1*. Student's *t* test was used to calculate p-values. Error bars denote mean ± SEM (*n* = 3). (F) Histological quantification of normal and cancer tissues. **, $P < 0.01$. N.S., not significant.

The Journal of Experimental Medicine

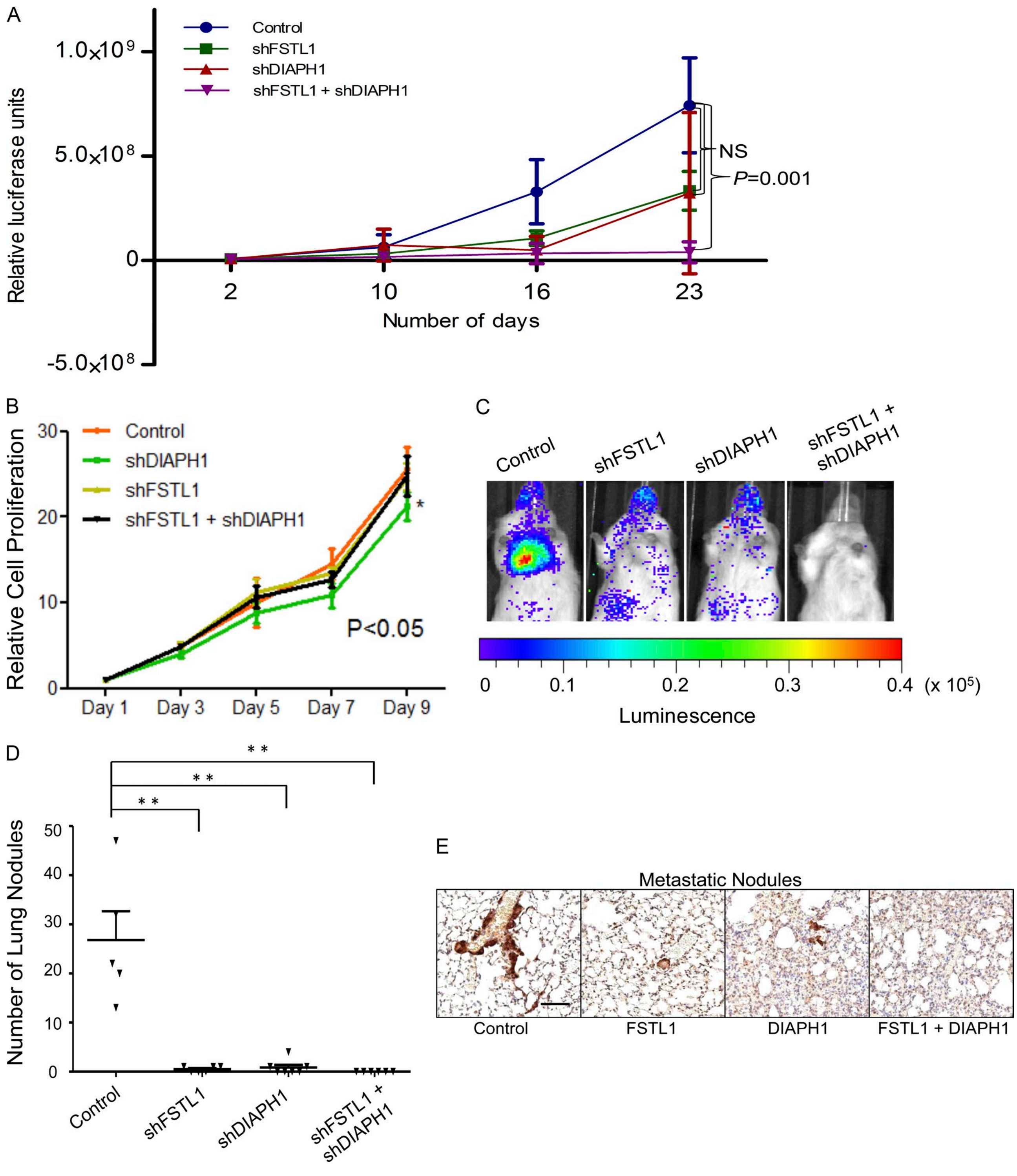

Figure S2. **FSTL1 and DIAPH1 promote metastatic colonization.** (A) Line graph representing the bioluminescence data from in vivo subcutaneous experiments. Error bars represent SD ($n = 5$). Statistical significance calculated by one-way analysis of variance with Bonferroni postcorrection. (B) Analysis of relative cell proliferation rate indicates no significant change upon knockdown of FSTL1 or DIAPH1 independently or in combination. Error bars represent SD. (C) Bioluminescence imaging of systemic metastasis by FaDu cells expressing luciferase reporter and control shRNA or shRNA against FSTL1 or DIAPH1 individually or in combination ($n = 7$). (D) Representation of the total number of metastatic foci in lung sections. **, $P < 0.001$. Error bars denote mean ± SEM. Statistical significance calculated by one-way analysis of variance with Bonferroni postcorrection. (E) Lungs were extracted on day 26 and stained for KRT5 expression; representative images of lung metastatic colonies shown. Bar, 100 µm.

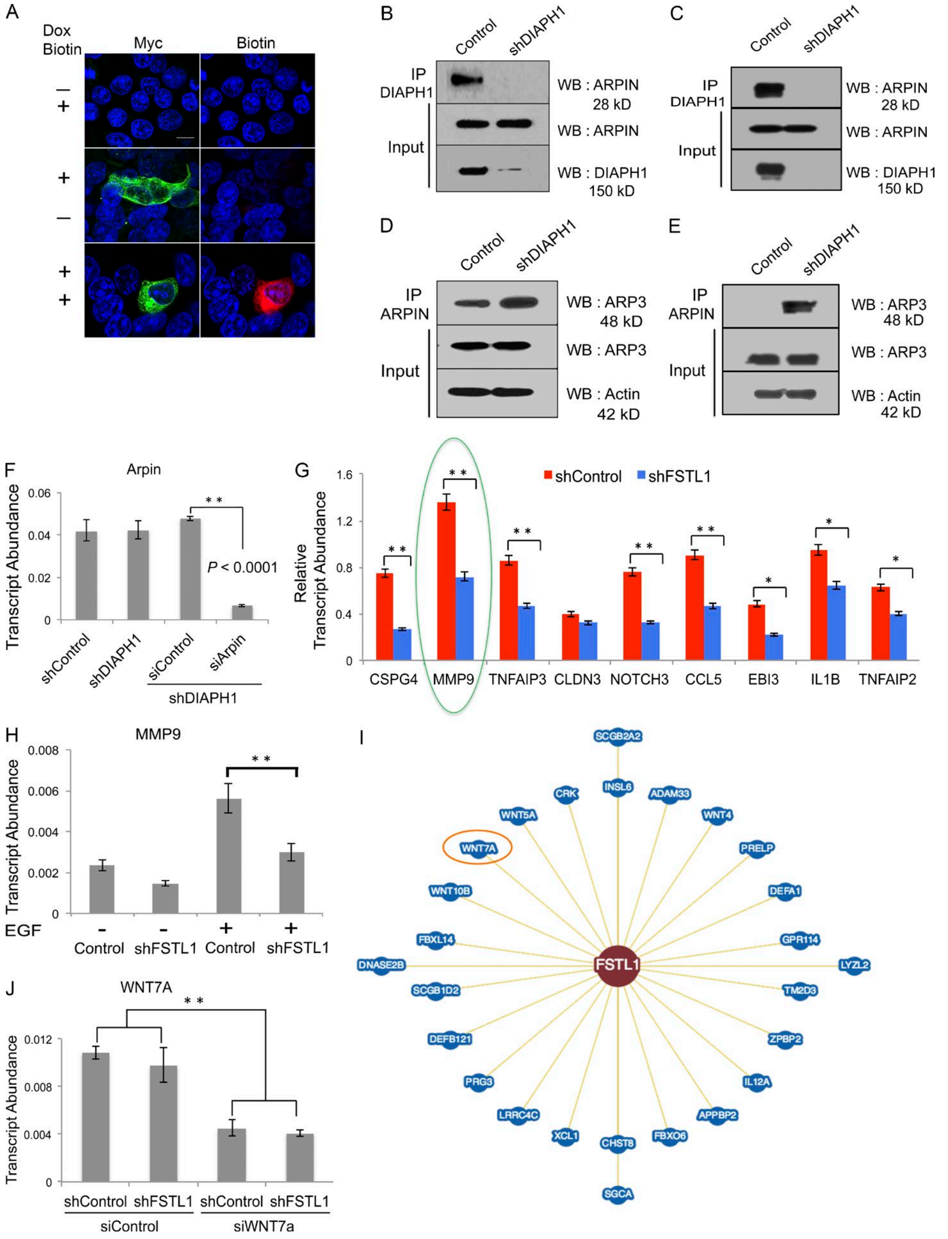

Figure S3. **Elucidation of underlying molecular mechanisms.** (A) Biotinylation of target proteins for BioID. 293T cells were transiently transfected with pTRIPZ vectors expressing DIAPH1 open reading frame, N-terminally fused with BirA*-myc tag under tet-inducible system. After transfection, cells were treated with 50 µM biotin, 10 µg/ml Dox, or both. BirA*-DIAPH1 fusion protein expression was probed using an anti-myc antibody, followed by species-specific secondary antibody coupled to Alexa Fluor 488. Biotinylation of target proteins was detected using streptavidin conjugated to Alexa Fluor 555. Bar, 10 µm. (B) DIAPH1 promotes directional persistence of migration. A253 cell lysates from control or shDIAPH1 cells were subjected to immunoprecipitation with anti-DIAPH1 antibody, and immunoprecipitates were probed for Arpin by Western blot analysis (top). Input cell lysates were probed for ARPIN (middle) and DIAPH1 (bottom). (C) Experiment from B repeated in FaDu cell line ($n = 3$). (D) A253 cell lysates from control or shDIAPH1 cells were subjected to immunoprecipitation with anti-Arpin antibody, and immunoprecipitates were probed for Arp3 by Western blot analysis (top). Input cell lysates were probed for Arp3 (middle) and β-actin (bottom; $n = 3$). IP, immunoprecipitation; WB, Western blotting. (E) Experiment from D repeated in FaDu cell line. (F) Histogram representing relative transcript abundance of Arpin upon DIAPH1 knockdown or simultaneous knockdown of both Arpin and DIAPH1 ($n = 3$). Error bar denotes SEM. Students *t* test was used for p-value calculation. (G) Validation of microarray data. Histogram representing relative transcript abundances (control vs. FSTL1 knockdown) of selected genes identified from microarrays (Fig. 4 D) and validated by qRT-PCR. Results show significant correlation with microarray data ($n = 3$). (H) Control or shFSTL1 cells were pretreated with EGF. Histogram represents the relative transcript abundance of MMP9 in these samples. Student's *t* test was used to calculate p-values, and error bars denote mean ± SEM. *, $P < 0.05$; **, $P < 0.001$. (I) BioGRID network analysis. Snapshot of proteins predicted to interact with FSTL1 protein from BioGRID v3.4.134 showing 26 unique interacting partners. (J) Relative levels of Wnt7a mRNA in A253 control or shFSTL1 cells transiently transfected with 50 nM control siRNA or siWnt7a. Error bars denote mean ± SEM ($n = 3$).
